# Frailty and functional status among older adults with cognitive impairment: data from the second wave of the FIBRA study

**DOI:** 10.1590/1980-5764-DN-2023-0051

**Published:** 2024-02-09

**Authors:** Beatriz Raz Franco de Santana, Flávia Silva Arbex Borim, Daniela de Assumpção, Anita Liberalesso Neri, Mônica Sanches Yassuda

**Affiliations:** 1 Postgraduate Program in Gerontology, Faculdade de Ciências Médicas, Universidade Estadual de Campinas, Campinas SP, Brazil.; 2 Gerontolgy, Escola de Artes, Ciências e Humanidades, Universidade de São Paulo, São Paulo SP, Brazil.

**Keywords:** Cognition, Aged, Frailty, Depression, Functional Status, Cognição, Idoso, Fragilidade, Depressão, Estado Funcional

## Abstract

Frailty is defined as a recognizable state of increased vulnerability resulting from age-associated decline of function in various physiological systems, such that the ability to deal with acute or everyday stressors is compromised.

**Objective:**

The aim of the study was to characterize the sample of older adults with cognitive impairment, according to the frailty status indirectly assessed by family members, other clinical and sociodemographic variables; and to assess the overlap of clinical conditions evaluated in this sample with cognitive impairment.

**Methods:**

Data were extracted from the follow-up database of the Frailty in Brazilian Older Adults (FIBRA) study (2016-2017). The sample consisted of 130 elderly people with cognitive impairment assessed by the Mini Mental State Examination (MMSE). The scores for the Clinical Dementia Scale (CDR), Cornell Scale for Depression in Dementia and Functional Activities Questionnaire were described. Frailty was indirectly measured through questions answered by family members about the five criteria that compose the frailty phenotype.

**Results:**

The sample consisted mostly of older women (n=91) with a mean age of 82.4 (SD=5.3) years, mean schooling of 3.3 years (SD=3.07), widowed (47.7%) and who lived with children and/or grandchildren (68%). More than half had multimorbidity (74.90%), 39.5% had depression symptoms suggestive of major depression, 57% had impaired functionality, 49.3% were frail, 37.6% pre-frail, and 13.10% robust.

**Conclusion:**

Among older adults with cognitive impairment, frailty and functional limitations are common.

## RESUMO.

A fragilidade é definida como um estado reconhecível de vulnerabilidade aumentada resultante do declínio da função associado à idade em vários sistemas fisiológicos, de modo que a capacidade de lidar com estressores agudos ou cotidianos fica comprometida.

**Objetivo:**

Caracterizar uma amostra de pessoas idosas com comprometimento cognitivo, segundo o estado de fragilidade, avaliado de forma indireta por familiares, assim como outras variáveis clínicas e sociodemográficas; e avaliar a sobreposição das condições clínicas avaliadas nesta amostra com o comprometimento cognitivo.

**Métodos:**

Os dados foram extraídos do banco de dados de acompanhamento do estudo Fragilidade em Idosos Brasileiros (FIBRA - 2016-2017). A amostra foi composta por 130 idosos com comprometimento cognitivo avaliado pelo Mini-Exame do Estado Mental (MEEM). Foram descritos os escores da Escala Clínica de Demência (CDR), da Escala Cornell de Depressão em Demência e do Questionário de Atividades Funcionais. A fragilidade foi mensurada indiretamente por meio de questões respondidas junto aos familiares sobre os cinco critérios que compõem o fenótipo de fragilidade.

**Resultados:**

A amostra foi composta em sua maioria por mulheres idosas (n=91) com idade média de 82,4 (DP=5,3) anos, escolaridade média de 3,3 anos (DP=3,07), viúvas (47,7%) e que viviam com filhos e/ou netos (68%). Mais da metade apresentava multimorbidade (74,90%), 39,5% apresentavam sintomas depressivos sugestivos de depressão maior, 57% tinham funcionalidade prejudicada, 49,3% eram frágeis, 37,6% pré-frágeis e 13,10% robustos.

**Conclusão:**

Entre idosos com alterações cognitivas, é comum a co-ocorrência de fragilidade e de limitações funcionais.

## INTRODUCTION

The number of people with dementia is increasing as the world’s population ages, leading older people to socioeconomic and health challenges^1^. With the increase in life expectancy and population aging, physical frailty comes to represent a risk factor for cognitive decline and dementia^2^.

Frailty is a syndrome characterized by increased vulnerability to stressors and decreased physiological reserves^3^. The frailty phenotype, based on data from epidemiological studies on cardiovascular^3^ and women’s health^4^, is linked to five criteria: weight loss, reduced handgrip strength, low gait speed, self-reported fatigue, and decreased physical activity level. Older people who do not have any of these characteristics are considered robust, those with one or two are considered pre-frail, and those with three or more are considered frail.

Besides worse health outcomes, frail older adults have worse cognitive performance than non-frail ones^5^ ^,^ ^6^ and tend to show faster cognitive decline and higher risk for dementia^7^. Some authors have argued that the simultaneous occurrence of frailty and cognitive impairment, in the absence of brain changes suggestive of dementia, should be regarded as the cognitive frailty syndrome^8^ ^,^ ^9^ ^,^ ^10^. The frailty syndrome can also lead to reduced healthy life expectancy and impaired autonomy and functionality^11^. Functionality is understood as the ability to carry out essential life activities, including taking care of oneself, living independently and performing activities that are important for quality of life^11^ ^,^ ^12^. Several international studies have associated frailty with cognitive and functional status^13^ ^,^ ^14^ ^,^ ^15^ ^,^ ^16^.

Another highly relevant factor related to frailty and cognitive impairment is the prevalence of depression, which ranges from around 10 to 20%^17^ ^,^ ^18^, among older adults. Cross-sectional studies^18^ observed the occurrence of frailty between 16% and 35% in elderly people with depression, and others reported a higher prevalence (up to 46.5%) of depression among frail older adults^19^. There is also an overlap of the clinical characteristics of both conditions, so that there is uncertainty as to the nature of frailty as a comorbidity, as a cause or as a consequence of depression. It is possible that both clinical conditions occur in individuals with high physical vulnerability. Studies indicate that both frailty and depression are risk factors for cognitive decline in the older population^20^.

In Brazil, the Frailty in Brazilian Older Adults (FIBRA) study aimed to investigate the relationships between frailty and demographic, socioeconomic, physical and psychological health variables in people aged 65 years or older. It was observed, in the baseline, that frail (9.1%) and pre-frail (51.8%) older adults corresponded to more than half of the sample^20^. The prevalence of frailty was comparable to that found in international studies, suggesting that senescence plays an important role in the frailty of older populations, despite different geographic and sociocultural contexts. After nine years, a follow up assessment was carried out and the present analyses originate from the second wave.

There are few studies describing frailty and associated conditions in samples with cognitive impairment, mainly in Brazil. It is an important contribution to examine the prevalence of frailty and clinical conditions that may overlap in a sample with cognitive impairment in the oldest old. Thus, the objective of the present study was to characterize the sub-sample of participants with cognitive impairment, in the follow-up study of the FIBRA study, according to their frailty status, indirectly assessed by family members, and other clinical variables, such as depression symptoms and changes in functionality, in relation to sociodemographic variables. Additionally, the objective was to evaluate the degree of overlap among the clinical conditions observed in this sub-sample with cognitive impairment.

## METHODS

### Study design

The data for the present analysis was originated from the second wave of the FIBRA study. The present study is descriptive and cross-sectional. The FIBRA study^21^ is a population-based study whose objective was to investigate associations between frailty and demographic, socioeconomic, health and psychosocial variables in Brazilian elderly aged 65 years or older. In the baseline assessment (2008-2009), carried out in seven Brazilian locations in different regions of Brazil, the elderly who met the eligibility criteria were invited by the recruiters to attend a 60-90 minute assessment session. Eligibility criteria were being 65 years or older and being a permanent resident in the location. Exclusion criteria, assessed during the first visit to the participant´s home, included noticeable cognitive impairment suggestive of dementia, severe sequels of stroke, Parkinson’s disease in a severe or unstable stage, and severe hearing or visual impairments, elderly who were confined to bed, in a terminal state, with cancer undergoing treatment. Further details on the baseline evaluation methods of the FIBRA study can be found in Neri et al.^21^

Nine years after the baseline assessment (2016-2017), a follow-up study was carried out^22^. The baseline eligibility and exclusion criteria were used once again. During the follow-up interview, the participants completed the MMSE to assess cognitive status. When participants had MMSE scores below education-adjusted cut off scores, the interview was interrupted after the anthropometric assessments and a family member (or another close informant) completed a protocol about the health status of the participant.

The follow-up study only included samples from two locations in the State of São Paulo, the city of Campinas and Ermelino Matarazzo (n=1,284). In the follow-up assessment, 549 elderly people (42.7% of the initial sample in both locations) were located and re-interviewed, 192 (14.9%) had died since the baseline and 543 (42.4%) were considered as lost samples (not found, refusal, withdrawal, risk for interviewers, exclusion criteria). Among those re-interviewed, 130 people had cognitive impairment assessed using the MMSE. The cut off scores for the MMSE were: 17 for the illiterate; 22 for seniors ranging between 1 and 4 years of schooling; 24 for those with between 5 and 8 years of schooling and 26 for those with 9 or more years of schooling. These cut off scores represent the averages reported by Brucki et al.^23^, for each educational level, minus one standard deviation.

The study sample consisted of 109 participants from Campinas (84%) and 21 (16%) from Ermelino Matarazzo. Among the 130 family members who answered the questionnaire, 23% were spouses, 33% were children and 23% were grandchildren. 70% of the family members lived in the same household as the participant.

### Instruments

For the present analysis, the following measures were selected: Clinical Dementia Rating (CDR)^24^ ^,^ ^25^, Cornell Scale of Depression in Dementia^26^ ^,^ ^27^, Pfeffer Functional Activities Questionnaire (FAQ)^28^ ^,^ ^29^, and an indirect measure of frailty based on in the perception of the informant^30^. The interviews were conducted by graduate and undergraduate students, who had undergone extensive training in the appropriate use of the measures described below.

The CDR is often used to stage cognitive changes, and the score varies between healthy (CDR=0), questionable dementia (CDR=0.5), mild dementia (CDR=1), moderate dementia (CD=2) and severe dementia (CDR=3). Questions of memory, orientation, judgment and problem solving, community activities, home and leisure, and personal care are assessed. In the FIBRA study, this measure was included to evaluate the degree of cognitive impairment.

The Cornell Scale for Depression in Dementia was developed to assess signs and symptoms of depression in people with cognitive disorders, consisting of 19 items with information obtained through interviews with the family member. Each item is rated for severity on a 0-2 scale (0=absent, 1=mild or intermittent, 2=severe). The item scores are summed, and values between 0 and 9 indicate absence of major depression, from 10 to 17 probable major depression, and scores from 18 points or more indicate major depression^26^. There are some studies that show the applicability of Cornell Scale in non-demented patients^31^ ^,^ ^32^.

The FAQ^28^ assesses performance in 11 instrumental activities of daily living, such as controlling financial activities and preparing a complete meal. There are six answer options: independence in the activity, completes the activity with difficulty, needs assistance, is not able to complete the activity. The minimum score on the scale is 0, and the maximum is 30.

The lower the overall score, the higher the level of independence in performing instrumental activities of daily living. In the present analysis, 5 points or more were considered indicative of functional limitation.

The indirect measure of frailty was obtained through dichotomous questions answered by family members about possible weight loss, reduced handgrip strength, walking speed, physical activity and fatigue in the last year. This questionnaire was previously validated in the SABE study^30^. The sensitivity and specificity to identify pre-frailty were 89.7% and 24.3%, respectively, and 63.2% and 71.6%, to identify frailty.

Questions were asked about the diagnosis (yes or no) of diseases in the previous year, in order to determine the presence of multimorbidity. As for the use of medications, the questions were whether they had been using drugs prescribed in the last 3 months by a doctor or taken them on their own. As for nutritional aspects, weight and height measurements were taken and the BMI was calculated.

The FIBRA baseline project was approved by the Research Ethics Committee of Universidade Estadual de Campinas (208/2007 and 907.575). The project for the FIBRA follow-up study was approved by the same institution (1.332.651 and 2.952.507). All participants signed a Free and Informed Consent Form.

### Data analysis

Descriptive statistics were performed to explore the data. Frequency, position and dispersion analyzes were selected. The Venn diagram was used to examine the co-occurrences between the studied conditions.

## RESULTS

Table 1 presents the main characteristics of the sample (n=130). In this, 70.7% were women (n=92); 31.5% were aged between 72 and 79 years; 35.3% between 80 and 84 years old; and 33% 85 years or older. The mean age was 82.47 (±5.3) years. Half of the participants were widowed and most lived with their children and/or grandchildren. About 19% of the participants were not educated and 15% had studied for 5 years or more. The average schooling of the sample was 3.23 (±3.07) years. Fifty-two percent were not responsible for supporting the family. The average family income was R$ 2,513.90 (±2,142.43), approximately two minimum wages.

**Table 1.** Sociodemographic characteristics of participants in the Frailty in Brazilian Older Adults study with scores below cut off scores on the Mini Mental State Examination.

Variable	n=130 (%)
Age (years)	
72-79	41 (31.5)
80-84	46 (35.4)
85 or more	43 (33.1)
Schooling (years)	
0	24 (18.4)
1-4	86 (66.2)
5+	20 (15.4)
Marital status	
Married	49 (37.7)
Single	12 (9.2)
Divorced	7 (5.4)
Widowed	62 (47.7)
Living arrangement	
With spouse	31 (23.0)
Alone	11 (9.0)
With other people	88 (68.0)

Table 2 presents the clinical data. It is observed that 75% had two or three chronic diseases, characterizing multimorbidity, 43% of the total number of participants reported the use of five or more medications. As for the BMI, 39% were eutrophic, while 20% were underweight and 41% were overweight. 78% percent reported having a diagnosis of hypertension and 32% of diabetes mellitus, and 83% reported using medication to treat these diseases.

**Table 2.** Clinical characteristics of participants from the Frailty in Brazilian Older Adults study with scores below the Mini Mental State Examination cut off scores (n=130).

Variable	n (%)
Number of chronic diseases (n=130)	
0	1 (0.7)
1	22 (15.8)
2	37 (26.6)
3	34 (24.4)
4 a 6	36 (25.8)
Polypharmacy* (n=116)	
No medications	66 (56.9)
5 or more	50 (53.1)
BMI (n=130)	
Eutrophy	51 (39.3)
Underweight	26 (20.2)
Overweight	53 (40.7)

Note: *the n is lower due to missing data (n=116).

In Table 3, on the Cornell Scale, 39.4% had scores suggestive of major depression and 27.1% had probable depression. On the CDR scale, approximately 75% of the parwticipants had scores higher than zero. The mean MMSE score was 17.4 points (±4.23). Regarding functionality, the average score on the FAQ was 9.32 (±9), and 57% of the sample had functional impairment (FAQ≥5). According to the relative’s assessment of frailty, 64 (49.3%) were frail, 49 (37.6%) were pre-frail and 17 (13.1%) were robust.

**Table 3.** Cognitive, mood, functionality and frailty characteristics of participants from the Frailty in Brazilian Older Adults study Study with scores lower than the Mini Mental State Examination cut off scores.

Variable	n (%)
Cornell scale* (n=109)	
Absent	33 (38.3)
Probable major depression	31 (27.2)
Major depression	45 (39.5)
Clinical dementia rating (n=130)	
None (0)	32 (24.6)
Questionable (0.5)	50 (38.5)
Mild (1)	22 (16.9)
Moderate (2)	16 (12.3)
Severe (3)	10 (7.7)
Functional Activities Questionnarie (Pfeffer)^†^ (n=128)	
Independent	55 (43.0)
Dependent	73 (57.0)
Frailty Measures (n=130)	
Robust	17 (13.1)
Pre-Frail	49 (37.6)
Frail	64 (49.3)

Note: *the n is lower in Cornell Scale due to missing data (n=109); ^†^the n is lower in Pfeffer Scale due to missing data (n=128).

**Figure 1.**
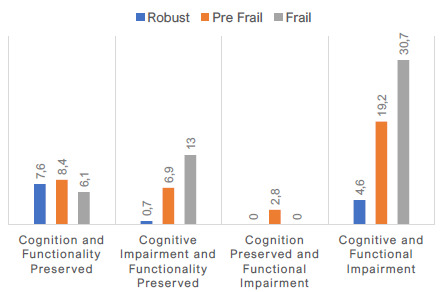
Data of cognition and functionality according to frailty.

**Figure 2.**
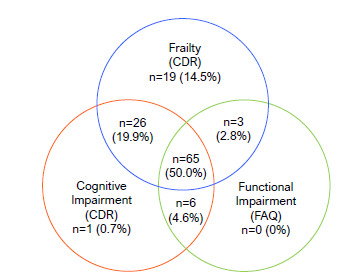
Venn Diagram with overlap of frailty, cognitive and functional impairment.

Among participants with normal cognition and functional status, 6.1% were frail (n=8), 8.4% pre-frail (n=11) and 7.6% robust (n=10); among those with impaired cognition and preserved functional status (a condition compatible with mild cognitive impairment), 13% were frail (n=17), 6.9% pre-frail (n=9) and 0.7% robust (n=1); among those with altered cognition and functional status (condition compatible with possible dementia) 30.7% were frail (n=40), 19.2% pre-frail (n=25) and 4.6% robust (n=6); and, among those with preserved cognition and altered functional status, no participant was classified as frail or robust, and 2.8% were pre-frail (n=3). Figure 1 summarizes the data, and the Venn diagram in Figure 2 describes the overlap between clinical conditions.

## DISCUSSION

The objective of the present analysis was to characterize participants with cognitive impairment in the follow-up assessment of the FIBRA study, according to sociodemographic and health variables. We also aimed to examine the co-occurrence of frailty, cognitive and functional impairment.

In the sample, there was a greater presence of women, mostly widows who lived with their children and/or other relatives or friends. Schooling was low, with 19% of people without schooling. 75% had multimorbidity. The prevalence of hypertension and diabetes was higher than in the general older population. The presence of symptoms suggestive of major depression was also observed in a high number of participants (39.5%), as well as functional impairment in more than half of the sample. On the other hand, 25% of participants, according to CDR data, had cognitive and functional preservation (CDR=0).

The sample has characteristics similar to those reported in the systematic review by Grande et al.^33^ on the co-occurrence of cognitive impairment, dementia and frailty, which included 13 articles. The average age of participants in the studies included in the review was 80 years, with an average follow-up of four to five years, a greater presence of women and an average education of seven to eight years. Schooling was higher than in the present study, as the articles originated from high-income countries in Europe. In Brazil, the study by César-Freitas et al.^34^, which followed older Brazilians in the city of Tremembé for five years to assess the incidence of dementia, revealed sociodemographic and health characteristics similar to those reported in our study.

Many studies have documented the association of frailty and worse cognitive performance. For instance, a meta-analysis compared the cognitive performance of non-frail older adults with pre-frail ones and non-frail with frail ones, and showed that statistically significant differences were found for both comparisons. Such findings, pooled over thousands of participants, indicated that physical frailty has implications for cognitive health^35^. Grande et al.^33^, in an additional systematic review and meta-analysis, reported that dementia incidence is significantly higher among older adults with cognitive impairment and frailty, in comparison to individuals who have only one of those conditions. In the present sample, with cognitive impairment in the MMSE, 37.6% were pre-frail and 49.3% were frail, suggesting there is a significant overlap between frailty and cognitive impairment.

Frailty in this sub-sample was assessed indirectly, that is, informed by family members. Based on these measures, we identified 49.3% of frail individuals and 37.6% of pre-frail individuals. The SABE^30^ study also evaluated frailty indirectly using the same scale and identified, in the city of São Paulo, a prevalence of 14% of frail and 54% of pre-frail individuals. However, the SABE study sample was not composed exclusively of people with cognitive impairment, as is the case of the present study. In Farías-Antunez et al.^36^, a Brazilian community-based study carried out in Pelotas, the Edmonton Frailty Scale was used in an adapted way, based on an interview with the caregiver, in order to assess the frailty of older participants who could not adequately answer the questions of the scale. The sample consisted mostly of women with low education, aged 75 years or older. A high presence of multimorbidity was observed (76%), and 13.8% were frail, while 63.9% were pre-frail. These data suggest that the high prevalence of frailty observed in the present study is possibly due to the presence of cognitive impairment in this sub-sample of FIBRA, and it is not only related to the fact that the measurement was obtained indirectly.

When we examined the overlap of frailty, cognitive (CDR) and functional impairments in the Venn diagram, we observed that participants with normal cognition (CDR=0 according to family members) and functional status were mostly pre-frail. Among those with altered cognition and preserved functional status (suggesting mild cognitive impairment) and those with altered cognition and functional status (suggesting dementia), most were frail. The Venn diagram demonstrates a high overlap of such conditions in this subsample. Single conditions (only frailty, cognitive or functional impairment) were rare (<10%).

In the present sample, functional status was altered in more than 50% of the participants. The study by Gontijo et al.^37^ showed that cognitive changes were associated with functional capacity and frailty, regardless of sex. In the study, it was observed that approximately 45% of the participants presented results suggestive of cognitive impairment and 24% presented functional incapacity. When compared to the present study, it is possible to observe that there is a significant number of participants with simultaneous impairment in cognition and functional status, with 37.4% of these also being frail and 29.0% pre-frail and/or robust. Such data show the frequent overlap of these conditions, which can be influenced by sociodemographic variables such as age, sex and education.

Data on depression symptoms were collected using the Cornell Scale, and 39.5% of the participants in this analysis had symptoms compatible with major depression. A narrative review^38^ analyzed 28 articles that studied the relationship between frailty and depression. The review highlights that the cooccurrence of both syndromes relates to worse health outcomes and higher vulnerability to negative events. Other authors have reported that frailty and depression may have overlapping characteristics, yet they constitute distinct constructs which may be bidirectionally associated^39^. Depressed patients with frailty may have higher mortality rates and fall risk due to use of antidepressants^39^.

These associations may also be observed among older adults without depression. In a Turkish study with 612 older participants without dementia and depression, frail and pre-frail individuals had higher Geriatric Depression Scale scores and lower cognitive performance^40^. Among 5,431 community-dwelling Chinese older adults^41^, pre-frail and frail participants had worse cognitive function and faster cognitive decline in eight years. Furthermore, it was observed that limitations in activities of daily living and depression symptoms mediated the association between physical frailty and cognitive function but did not affect its rate of decline^41^. Therefore, depression symptoms may alter the impact of frailty on cognitive performance. These studies illustrate that frailty, depression and cognitive performance are interdependent.

Among the limitations of the present study, the size of the sample of participants in the FIBRA follow-up study stands out, since there was a significant sample loss after nine years. It is worth considering that we used an indirect measure of frailty with responses from the family member who lived with and/or cared for the participant. Although this frailty instrument presents adequate sensitivity and specificity in determining frailty, the psychometric parameters for the individual frailty components fall short from the ideal, as previously reported^30^. This aspect of the instrument may have biased results; however, the present analyses were limited to frailty status. We also acknowledge that there is some degree of overlap between the assessed domains (e.g., cognitive impairment, dementia and depressive symptoms). Additionally, we also highlight that there is limited data concerning the informants who responded to the family member’s protocol.

In conclusion, we observed that among participants who presented cognitive impairment, frailty and functional alterations were highly prevalent. FutureBrazilian studies should investigate longitudinally the predictive factors of cognitive impairment and dementia, including frailty, depression and variables related to lifestyle. In addition, more studies should investigate the physical and mental health conditions of participants with cognitive impairment in epidemiological studies. Present findings suggest that this subsample is more prone to present multimorbidities and they are most likely to present worse health outcomes.
